# Metagenomic Next-Generation Sequencing (mNGS) for the Timely Diagnosis of Carbapenem-Resistant Klebsiella pneumoniae in Leukemia Patients

**DOI:** 10.1155/2022/6957028

**Published:** 2022-12-08

**Authors:** Huan Tao, Fang Hua, Yingying Chen, Yongqian Jia

**Affiliations:** ^1^Department of Hematology, West China Hospital of Sichuan University, Chengdu, China; ^2^Department of Hematology, Zigong First People's Hospital, Sichuan, China; ^3^Department of Oncology, Sichuan Cancer Hospital, Chengdu, China

## Abstract

This report shows the contribution of metagenomic next-generation sequencing (mNGS) as an alternative to challenging diagnostic infection in immunosuppressed individuals. Herein, we report two leukemia patients who developed severe infections due to carbapenem-resistant Klebsiella pneumoniae (CrKP). The mNGS can be strongly recommended as an alternative investigation for patients who are at high risk of infection without positivity on body fluid culture. This can provide the opportunity for adequate therapy.

## 1. Introduction

Bloodstream infections (BSIs) caused by carbapenem-resistant Klebsiella pneumoniae (CrKP) have received increasing attention as a timely diagnosis and therapeutic issue [1, 2], especially for patients with a hematological malignancy [3, 4]. Patients with CrKP infection have a poor prognosis and a high mortality rate [5]. Thus, timely diagnosis and treatment are very important.

Routine microbiologic testing is time-consuming and not sufficient to diagnose several infectious complications in hematological patients, and only 50% of blood cultures from septic shock patients are positive [6]. Metagenomic next-generation sequencing (mNGS) has been applied in BSIs with the advantages of a rapid and high positive rate in the detection of pathogens in patients with severe sepsis [7, 8]. Here, we present two severely immune-compromised leukemia patients illustrating the timely diagnosis by mNGS and therapy caused by CrKP.

## 2. Case Presentation

### 2.1. Case 1

**Figure 1:**
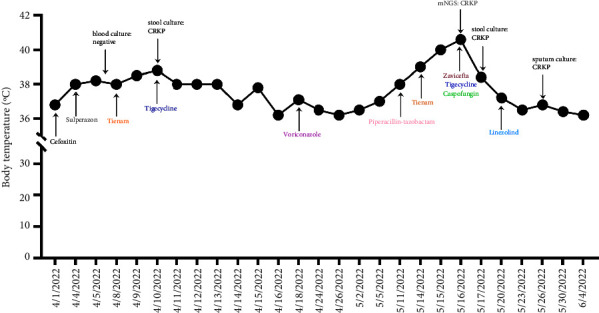
Anti-infective therapy of case 1.

A 24 year-old young man was admitted to our hospital and presented with fatigue, headache, palpitations, and tinnitus. After the examination, he was diagnosed with acute myelogenous leukemia. He achieved complete response (CR) following induction and consolidation chemotherapy. He developed febrile neutropenia in the first chemotherapy cycle. Patch shadows and strip shadow were observed by computed tomography (CT) scan. Blood cultures were negative. Considering infection, cefoxitin, sulperazon, amikacin, tienam, and vancomycin were given successively. However, the patient still had recurrent fever, accompanied by new physical examination showing abdominal tenderness, rebound pain, and muscle tension. A CT scan showed signs of peritonitis. Blood culture was still negative, and the serum (1,3)-*β*-D glucan (G-test) and galactomannan (GM test) were negative. *Klebsiella pneumoniae* was found in stool culture as carbapenem-resistant Enterobacterales (CRE) colonization. Then tigecycline was administered according to the drug sensitivity test. Voriconazole was given for empirical antifungal therapy. After treatment, the patient's temperature returned to normal, and his abdominal pain improved significantly. After the second chemotherapy, he developed fever and neutropenia with laryngeal pain at first, followed by pharyngeal swelling congestion, tonsil I-II degree of swelling, abdominal tenderness, rebound pain, and muscle tension. Blood culture was negative. Piperacillin-tazobactam was given for anti-infection, but symptoms were not relieved. Two positive stool cultures suggested that CrKP and tigecycline were upgraded to anti-infection. However, a daily fever continued, and the highest temperature was 40°C. The pain in the throat and abdomen was significantly worse than before. Laboratory investigation showed amounts of white blood cell (WBC) count of 0.16 × 10^9^/L (normal range, 3.5–9.5) and neutrophils count of 0 (normal range, 1.8–6.3). C-reactive protein (CRP) was 284.00 mg/L (normal range, <5), interleukin-6 (IL-6) was 378.00 pg/ml (normal range, <7), and procalcitonin (PCT) was 20.00 ng/mL (normal range, <0.046). A blood sample was collected for mNGS perform. The test was performed by Simcere. Sequencing technology based on the Illumina second-generation high-throughput sequencing platform was used to extract the nucleic acid sequences of all microorganisms in the samples for sequencing. Then, the species information of pathogenic microorganisms was analyzed by comparing the special pathogen database and intelligent bioinformatics algorithm. This method directly sequenced all nucleic acids in the sample without culture and prediction of pathogenic microorganisms. A total of 19,918 pathogens were included in Simcere pathogen_database V2.1. The database is regularly updated to provide a comprehensive collection of endemic and/or proprietary genomes in China. Fortunately, mNGS revealed the presence of *K. pneumoniae* (63,427 unique reads) with drug resistance genes, such as blaCTX-M (14 reads), blaTEM (12 reads), and blaKPC (10 reads). Referring to the results, zavicefta (ceftazidime-avibactam) was given immediately. After treatment, the patient's temperature returned to normal, and the symptoms disappeared. The anti-infective therapy is detailed in Figure 1, and more information is detailed in Supplementary Figures 1 and 2. The human leukocyte antigen match between the patient and his sister is identical. Currently, he is awaiting allogenic hemopoietic stem cell transplantation.

### 2.2. Case 2

**Figure 2:**
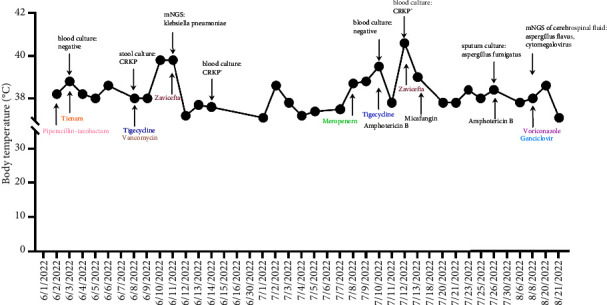
Anti-infective therapy of case 2.

A 54 year-old male patient was admitted to our hospital and presented with sore throat, lymph node growth, and abdominal pain. After the examination, he was considered to be diagnosed with acute monocytic leukemia and achieved CR after induction and consolidation chemotherapy treatment. The patient developed febrile neutropenia after the first chemotherapy. He was given piperacillin-tazobactam and Tienam but still had recurrent fever. Blood culture remained negative while the stool culture indicated CrKP, and then antibiotics were adjusted to tigecycline and vancomycin. After that, the patient developed fever again at 39.8°C and blood pressure of 65/40 mmHg. Septic shock was considered. Blood samples were collected for mNGS perform and blood culture. Sixteen hours later, mNGS revealed the presence of *K. pneumoniae* (225 unique reads) without drug resistance genes. Zavicefta (ceftazidime-avibactam) was given immediately for anti-infection. Four days later, blood culture showed CrKP. After treatment, the patient's body temperature and blood pressure returned to normal. One month after the consolidation chemotherapy, the patient complained of headache. CT scan showed scattered low-density shadows in the left part of the brainstem and the left cerebellar hemisphere. Flow cytometry of cerebrospinal fluid showed no abnormal primitive cells. Routine cerebrospinal fluid and biochemical analyses found no obvious abnormalities; IL-6 1487.00 pg/mL, interleukin-10 65.74 pg/mL. Cerebrospinal fluid was detected by mNGS, and *Aspergillus flavus* (62 unique reads) and *cytomegalovirus* (218 unique reads) were detected, while the culture was negative. Considering an intracranial fungal infection, the patient was treated with voriconazole for antifungal therapy. The headache was obviously relieved after treatment. Oral isavuconazonium was continued after discharge from the hospital. The anti-infective therapy is detailed in Figure 2 and more information is detailed in Supplementary Figures 3 and 4.

## 3. Discussion

Our patients' clinical course proved the challenge of infection management in leukemia patients. Our cases highlight the CRE colonization and timely diagnosis and therapy of CrKP in leukemia patients.

BSIs caused by Enterobacterales have become increasingly life-threatening [1]. *K. pneumoniae* is an opportunistic pathogen in cancer patients suffering from chemotherapy that causes infections, and the majority of hypervirulent *K. pneumoniae* is antibiotic-susceptible, but the combination of multidrug resistance and enhanced virulence has the potential to cause the next clinical crisis, such as the CrKP [9]. CrKP is highly sensitive to tigecycline, Zavicefta, and polymyxin, and some cases were reported to be resistant to Zavicefta [10].

CRE intestinal colonization can increase the incidence of CRE infection in patients with acute leukemia [11]. CrKP accounts for approximately 60% of CRE infections in China [12]. Approximately, 65% of leukemia patients undergoing induction chemotherapy were colonized with bacteria, and *K. pneumoniae* was the most common [13]. In our case 1 and case 2, CrKP intestinal colonization was all founded, and tigecycline was given timely, but the body temperature increased. Then, the blood culture or mNGS was subsequently arranged subsequently, and the bacteremia was confirmed in our two cases. This fact could remind clinicians to think about whether the colonized CrKP caused bacteremia when the anti-infection effect was not significant.

Routine microbiologic testing is frequently insufficient to detect uncommon pathogens, is time-consuming, and has a low positive rate [6]. While mNGS is a high-throughput, has short turnaround time, and has the accurate sequencing method that directly extracts all microbial DNA from clinical samples rather than cultures from the samples [8]. The mNGS test revealed *Candida tropicalis* in liver biopsy while the culture remained negative [14]. In case 2, the mNGS test revealed the presence of *K. pneumoniae* after 16 hours while blood culture showed CrKP after 4 days. We administered the Zavicefta immediately for anti-infection according to the mNGS results, and hyperthermia was markedly reduced. In addition, after excluding the infiltration of the leukemic cells in the central nervous system, *Aspergillus flavus* and *cytomegalovirus* were detected by mNGS with low reads in the cerebrospinal fluid, and prompt therapy obviously relieved the patient's headache. Thus, mNGS could be a powerful tool to supplement blood culture. Based on our cases, mNGS techniques can be applied in parallel with classical diagnostic techniques to have a double check of the results and to confirm a diagnosis with certainty. We strongly recommended mNGS as an additional investigation for frail patients for timely diagnosis of the infections and provided the opportunity for adequate therapy.

In conclusion, colonized AML patients were at high risk of CrKP bacteremia, and timely diagnosis and treatment were effective in improving survival. For high-risk hematological patients colonized by CrKP, the occurrence of a CrKP bacteremia should be strongly suspected when febrile neutropenia occurs. In addition, mNGS might provide faster and more accurate information for timely diagnosis than routine microbiologic testing. mNGS can be strongly recommended as an alternative investigation for patients who are at high risk of infection without positivity on body fluid culture. This might provide valuable suggestions for the clinical management of leukemia patients receiving regular chemotherapy.

## Data Availability

All data are available within the article.

## Abbreviations

mNGS: Metagenomic next-generation sequencing
CRE: Carbapenem-resistant Enterobacterales
CrKP: Carbapenem-resistant Klebsiella pneumoniae
BSIs: Bloodstream infections
CR: Complete response
CT: Computed tomography
WBC: White blood cell
CRP: C-Reactive protein
PCT: Procalcitonin
G-test: Serum(1,3)-*β*-D glucan
GM test: Galactomannan.

## Supplementary Materials

Supplementary Materials Supplementary Figure 1: The change trend of WBC, neutrophils, and blast cells in case 1 during the anti-infective therapy. Supplementary Figure 2: The change trend of body temperature, CRP, PCT, and IL-6 in case 1 during the anti-infective therapy. Supplementary Figure 3: The change trend of WBC, neutrophils, and blast cells in case 2 during the anti-infective therapy. Supplementary Figure 4: The change trend of body temperature, CRP, PCT, and IL-6 in case 2 during the anti-infective therapy.
