# Supplementary figures and images for: Metagenomic Next-Generation Sequencing (mNGS) for the Timely Diagnosis of Carbapenem-Resistant Klebsiella pneumoniae in Leukemia Patients
Source: Case Rep Infect Dis. 2022 Dec 8;2022:6957028. doi: 10.1155/2022/6957028 (PMC9754844; doi:10.1155/2022/6957028)

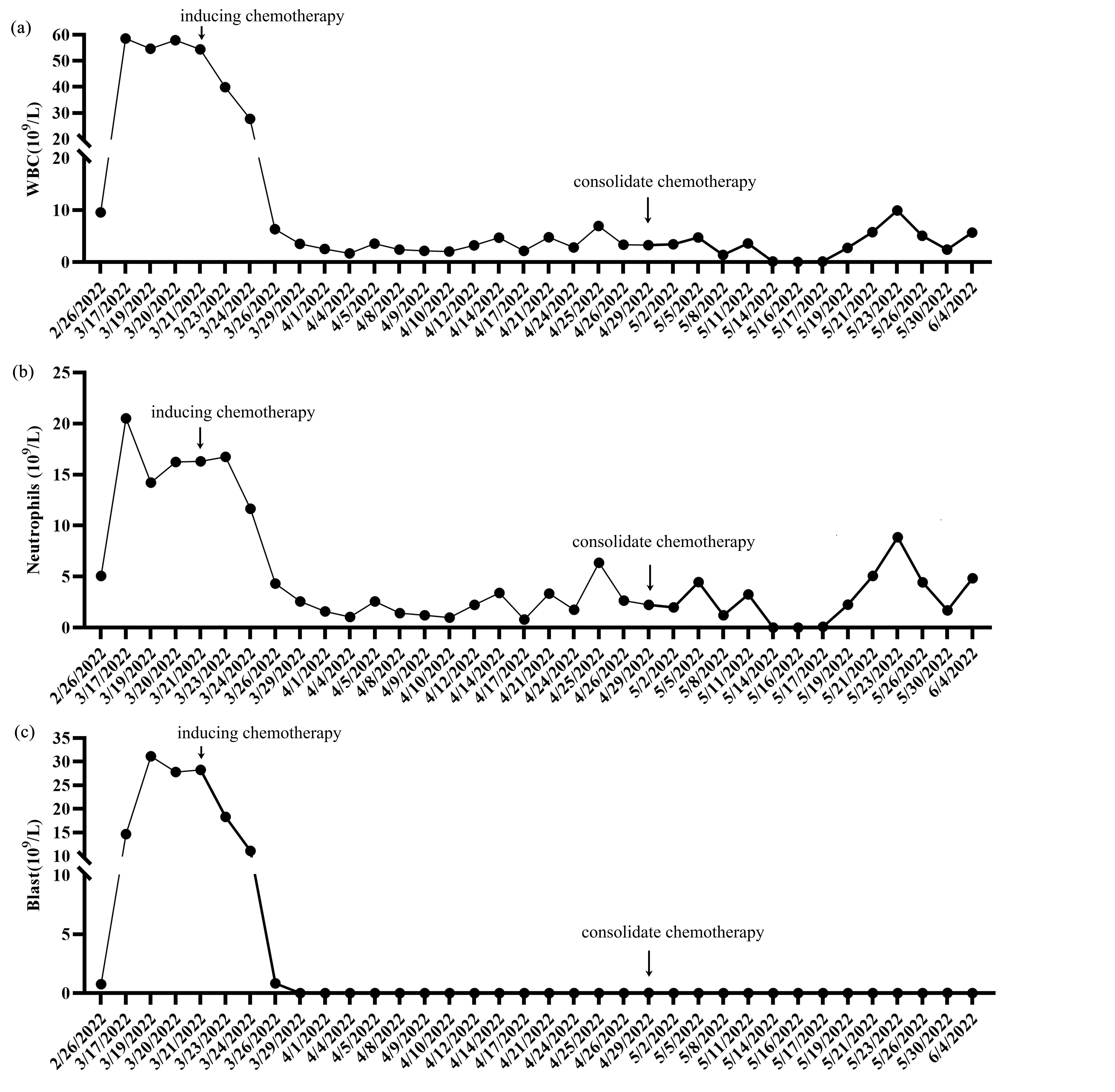

Supplement: Supplementary Materials — Supplementary Figure 1: The change trend of WBC, neutrophils, and blast cells in case 1 during the anti-infective therapy. Supplementary Figure 2: The change trend of body temperature, CRP, PCT, and IL-6 in case 1 during the anti-infective therapy. Supplementary Figure 3: The change trend of WBC, neutrophils, and blast cells in case 2 during the anti-infective therapy. Supplementary Figure 4: The change trend of body temperature, CRP, PCT, and IL-6 in case 2 during the anti-infective therapy. [file 6957028.f1.zip › Supplementary figure 1 (1).jpg]

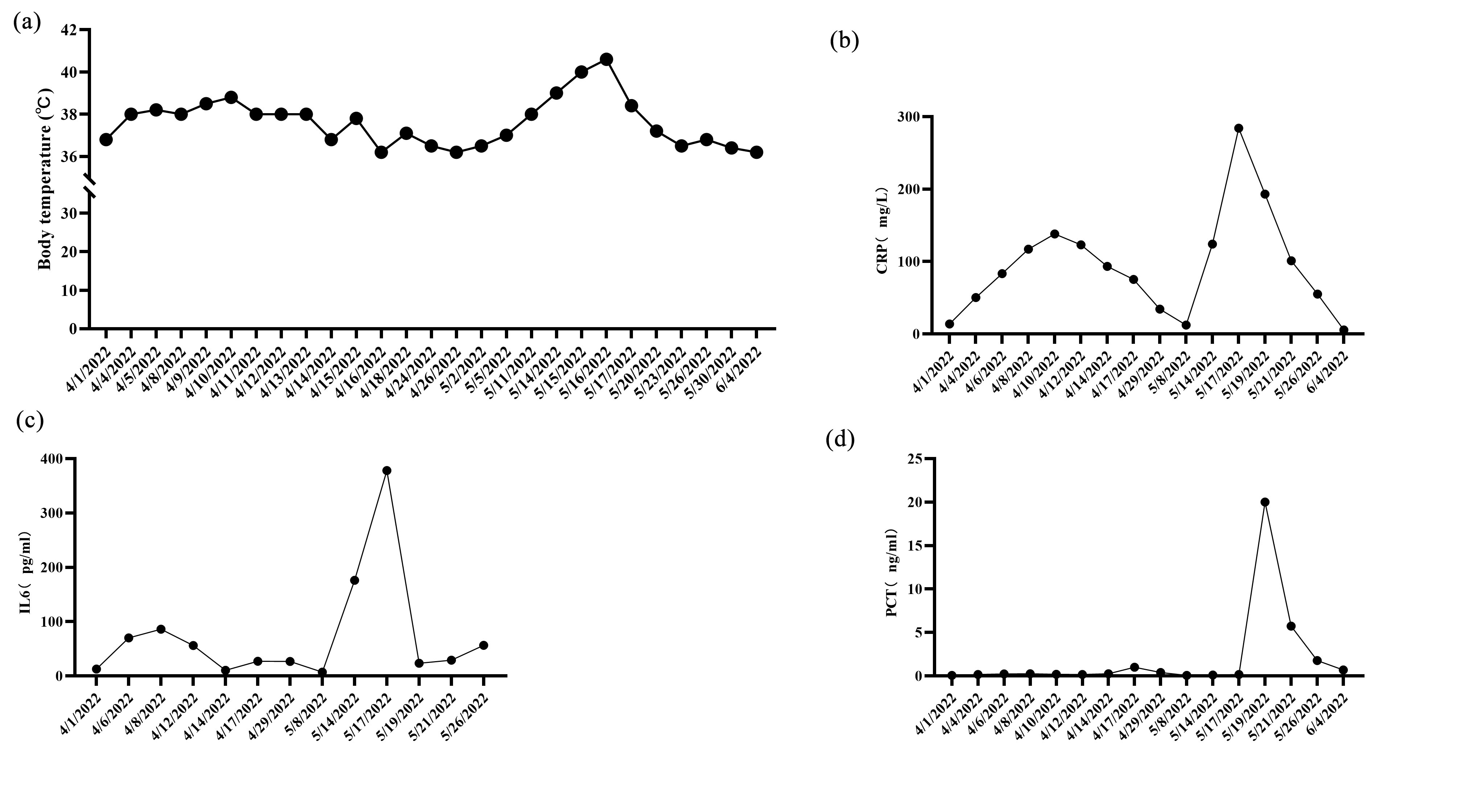

Supplement: Supplementary Materials — Supplementary Figure 1: The change trend of WBC, neutrophils, and blast cells in case 1 during the anti-infective therapy. Supplementary Figure 2: The change trend of body temperature, CRP, PCT, and IL-6 in case 1 during the anti-infective therapy. Supplementary Figure 3: The change trend of WBC, neutrophils, and blast cells in case 2 during the anti-infective therapy. Supplementary Figure 4: The change trend of body temperature, CRP, PCT, and IL-6 in case 2 during the anti-infective therapy. [file 6957028.f1.zip › Supplementary figure 2 (1).jpg]

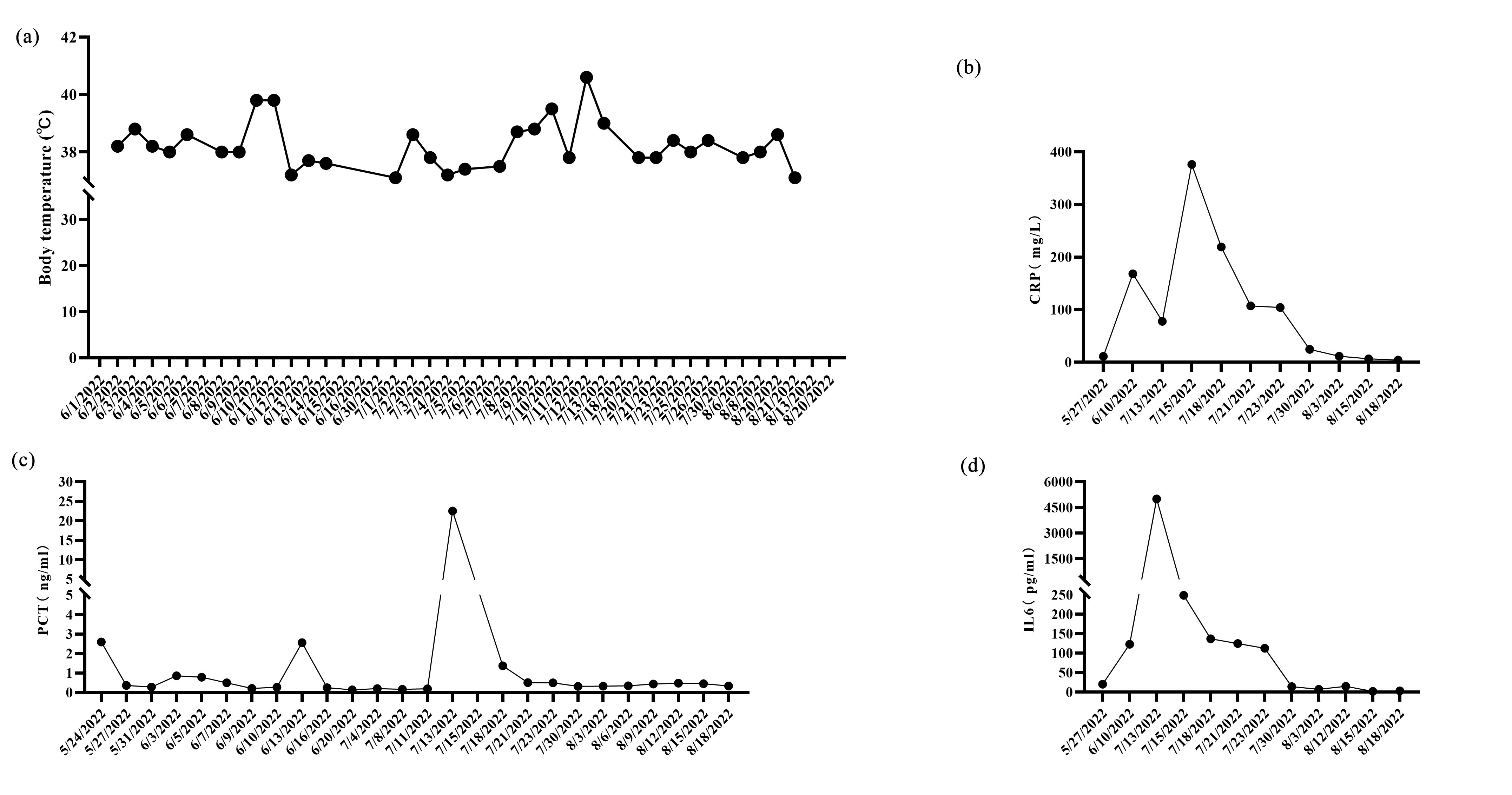

Supplement: Supplementary Materials — Supplementary Figure 1: The change trend of WBC, neutrophils, and blast cells in case 1 during the anti-infective therapy. Supplementary Figure 2: The change trend of body temperature, CRP, PCT, and IL-6 in case 1 during the anti-infective therapy. Supplementary Figure 3: The change trend of WBC, neutrophils, and blast cells in case 2 during the anti-infective therapy. Supplementary Figure 4: The change trend of body temperature, CRP, PCT, and IL-6 in case 2 during the anti-infective therapy. [file 6957028.f1.zip › Supplementary figure 4 (1).jpg]
